# Functional and *in silico* Characterization of Neutralizing Interactions Between Antibodies and the Foot-and-Mouth Disease Virus Immunodominant Antigenic Site

**DOI:** 10.3389/fvets.2021.554383

**Published:** 2021-05-07

**Authors:** Ruben Marrero Diaz de Villegas, Cristina Seki, Nora M. Mattion, Guido A. König

**Affiliations:** ^1^Instituto de Agrobiotecnología y Biología Molecular, Instituto Nacional de Tecnología Agropecuaria, Consejo Nacional de Investigaciones Científicas y Tecnológicas, Buenos Aires, Argentina; ^2^Centro de Virología Animal, Consejo Nacional de Investigaciones Científicas y Tecnológicas, Universidad Abierta Interamericana, Buenos Aires, Argentina

**Keywords:** FMDV, neutralizing antibody, peptide ELISA, functional epitope, structure modeling, docking

## Abstract

Molecular knowledge of virus–antibody interactions is essential for the development of better vaccines and for a timely assessment of the spread and severity of epidemics. For foot-and-mouth disease virus (FMDV) research, in particular, computational methods for antigen–antibody (Ag–Ab) interaction, and cross-antigenicity characterization and prediction are critical to design engineered vaccines with robust, long-lasting, and wider response against different strains. We integrated existing structural modeling and prediction algorithms to study the surface properties of FMDV Ags and Abs and their interaction. First, we explored four modeling and two Ag–Ab docking methods and implemented a computational pipeline based on a reference Ag–Ab structure for FMDV of serotype C, to be used as a source protocol for the study of unknown interaction pairs of Ag–Ab. Next, we obtained the variable region sequence of two monoclonal IgM and IgG antibodies that recognize and neutralize antigenic site A (AgSA) epitopes from South America serotype A FMDV and developed two peptide ELISAs for their fine epitope mapping. Then, we applied the previous Ag–Ab molecular structure modeling and docking protocol further scored by functional peptide ELISA data. This work highlights a possible different behavior in the immune response of IgG and IgM Ab isotypes. The present method yielded reliable Ab models with differential paratopes and Ag interaction topologies in concordance with their isotype classes. Moreover, it demonstrates the applicability of computational prediction techniques to the interaction phenomena between the FMDV immunodominant AgSA and Abs, and points out their potential utility as a metric for virus-related, massive Ab repertoire analysis or as a starting point for recombinant vaccine design.

## Introduction

FMD is a highly infectious animal disease affecting over 70 wild and domestic cloven-hoofed species such as cattle and swine (1). The etiological agent is FMDV, an Aphthovirus from the Picornaviridae family (single-stranded positive-sense RNA genome of ~8 kb) (2). Direct production losses and international trade restrictions in endemic countries, as well as the high cost of FMD control, are a major problem for governments and producers (1).

The humoral immune response has a fundamental role in the protection against FMDV. Neutralizing antibodies (nAbs) recognize functional regions at the viral capsid (antigenic sites, AgS) (3). The FMDV antigenic site A (AgSA) includes the key virus recognition motif (an RGD amino acid triad) for its major host cellular receptor (αVβ6 integrin). AgSA is located on capsid protein 1 (VP1) and triggers the main humoral neutralizing response (4).

Abs are also valuable biotechnology reagents and key molecules for FMDV diagnosis and surveillance (5). Recent FMDV publications have reported basic and applied Ab studies (6) by harnessing a wider sampling tool like next-generation sequencing (NGS), although a few explored the *in vivo* antibody (or immune receptor) repertoire (7–9). Using this approach, a broad neutralizing antibody was successfully obtained through single B-cell isolation from peripheral blood mononuclear cells of sequentially immunized bovines (10) to improve FMDV diagnostic tests. Other studies have focused on the molecular structure of Ag–Ab interactions of FMDV of serotype C. For example, Verdaguer et al. resolved the structure of a nAb interacting with a peptide equivalent to the AgSA of FMDV by X-ray diffraction analyses and characterization at quasi-atomic level (2.3 Å) (11, 12). A second serotype C Ab-FMDV structure encompassed a Cryo-EM reconstruction of Fab-capsid interactions at 30-Å resolution (13). Recently, another group reported the sequence and molecular modeling of a serotype O mAb (6).

The future for Ab knowledge and veterinary vaccinology applications is undoubtedly promising at the convergence of novel sequencing platforms and bioinformatic tools to explore the cattle and swine immunoglobulin repertoire and Ab structural information (14). Therefore, the Ag–Ab complex modeling is crucial. Herein, we obtained the Fab (antigen-binding fragment) sequence of two site A-specific mAbs of serotype A and characterized their functional epitopes through a peptide ELISA. Finally, we implemented a protocol for modeling the Ab molecular structure as well as their specific interaction with viral Ag at AgSA, based on homolog (X-ray diffraction) structures available for serotype C. Our research demonstrated that it is feasible to model the molecular structure of FMDV antibodies and the topology of Ag–Ab interactions as well as to predict the influence of mutations on those interactions.

## Materials and Methods

### Antibodies and Foot-and-Mouth Disease Virus (FMDV) Peptide Antigen

We developed and supported the modeling protocol by reviewing the mAb 4C4 complex and applying it to novel anti-serotype A mAbs. Published data using mAbs to study FMDV Ag–Ab interaction for their different capsid antigenic sites, including high atomic resolution structures, had only been obtained for the AgSA of serotype C virus (mAb 4C4). Sequence and atomic coordinate data for experiments including the mAb 4C4 (anti-FMDV of serotype C) were obtained from the protein data bank accession, pdb 1EJO (11, 12). Pdb 1EJO describes the interaction complex between mAb 4c4 and AgSA peptide from FMDV strain C-S8c1 (a biological clone derived from FMDV isolate C1 Sta Pau Sp/70) (15).

mAbs 1E12 and 4A2 (IgM and IgG, respectively) against a serotype A FMDV strain (A24Cruzeiro) were obtained from a previous study (16, 17). Both mAbs recognize epitopes at the AgSA of VP1 protein (17).

Fifteen peptides were designed to be used in ELISA (see the *Immunoassays* section) with point mutations designed according to the aa variability found at different positions in several FMDV strains (Supplementary Table 1) serologically sampled at the Centro de Virología Animal–CEVAN (16, 17). The 20-aa long, N-terminal biotinylated, peptides comprised the RGD region of the GH loop at VP1. The negative control peptide (ID 15), included the RGD motif, but with multiple mutations relative to the wt peptide (ID 14). The peptides were prepared and used according to the manufacturer's instructions (JPT Peptide Technologies).

### Immunoassays

The relative binding affinity between mAbs 1E12 or 4A2 and 15 single mutated antigens (single aa-substituted synthetic peptides) was estimated by two different immunoassays (ELISA) (18, 19).

A competitive peptide ELISA was implemented for mAb 4A2 (monomeric IgG) since this mAb's paratopes were effectively peptide inhibited. A similar peptide inhibition could not be achieved for mAb 1E12, probably due to its pentameric IgM form. Therefore, a sandwich ELISA was developed to assess the peptide interactions instead. ELISA results were normalized by the wt peptide values at each ELISA; however, a rigorous quantitative comparison cannot be established.

For the mAb 4A2 competitive ELISA, 96-well plates (Maxisorb NUNC Fisher) were coated with 15 ng of wt peptide and incubated overnight at 4°C (P1 plates). In a second set of control plates (P2), all peptides (wt and mutated versions) were tested simultaneously by three independent reactions containing a fixed non-saturating dilution of mAb 4A2 and three different amounts of peptide (1,350, 450, and 150 ng). After incubation (2 h at 37°C), peptide–Ab reactions were transferred from P2 to P1 plates and incubated for 1 h at 37°C. Next, plates were washed and further incubated with an anti-mouse IgG-HRP-conjugated mAb (HRPO Sigma) for 1 h at 37°C. After extensive washing with 0.05% Tween 20–0.1% bovine serum albumin in PBS, TMB substrate (3,3′, 5,5′-tetramethylbenzidine; BD OptEIA) was added to the plate and the resulting OD (optical density) was measured at 450 nm (Thermo Scientific Multiskan® Spectrum) to analyze Ag–Ab interactions. The background values, obtained with no mAb and synthetic peptide, were subtracted for the analysis, and a relative inhibitory concentration (IC50) was determined as the mass of each mutated peptide that causes 50% of binding inhibition relative to the homologous wt peptide (11).

A sandwich ELISA was implemented for the mAb 1E12. In this immunoassay, 96-well ELISA plates were pre-coated (16 h at 4°C) with a fixed 2.5 μg of avidin (NeutrAvidin Invitrogen®), which assured a homogenous capture of the 15 sampled biotinylated peptides (including wt). The procedure consisted of four peptide masses (4,000, 1,333, 444, and 148 ng) for each peptide and incubations for 2 h at 37°C, all in triplicate. Next, mAb 1E12 was added at a fixed dilution per well, followed by the addition of an anti-IgM-HRPO secondary mAb (Axell) and further incubation for 1 h at 37°C. Absorbance was read as described above, and the interaction was quantified as half-maximal effective concentration (EC50), referred to as the concentration of peptide that induces a response halfway between baseline and maximum after a given exposure time.

A qualitative comparison of both ELISA data was performed by defining a relative interaction index 50 (IIR50) according to a settled equivalence between IC50 and the inverse of EC50, relative to the same wt peptide.

### Cloning Foot-and-Mouth Disease Virus mAbs Coding Sequence

The molecular cloning of the FMDV neutralizing coding sequences of the evaluated antibodies (4A2 and 1E12) was carried out from mAb-producing hybridoma cell lines (16), without any prior information on their nucleotide or aa sequence (20). This procedure uses the polymerase chain reaction (PCR) to amplify the sequence of variable Ab regions based on the conservation of its flanking regions (21) and a canonical set of degenerate primers capable of amplifying most of the V regions of murine Abs (22).

Briefly, ~1–2 million mAb-secreting hybridoma cells were harvested and homogenized. Subsequently, total RNA from these cells was isolated using the RNeasy Mini Spin kit according to the manufacturer's instructions (QIAGEN). The cDNA was produced by reverse transcription (RT-PCR) using 10 μg of the RNA template, a 15-base oligo-dT primer, and SuperScript II reverse transcriptase enzyme (Invitrogen). The reactions were incubated at 42°C for 1 h, followed by inactivation of reverse transcriptase at 70°C for 15 min. The resulting cDNA was used as a template for PCR amplification using Taq polymerase (Invitrogen) and various 5′ and 3′ primers specific for VH and VL genes specified in the manual “Current Protocols in Immunology” (20). The PCR reactions were incubated at 94°C for 2 min, followed by 30 cycles of 96°C for 15 s, 56°C for 30 s, and 72°C for 2 min and a final extension at 72°C for 10 min. PCR products of the appropriate size (350–500 base pairs, purified from agarose gels) were cloned into the Invitrogen vector pCR2.1-TOPO-TA™ and transformed into *Escherichia coli* BL21 cells.

Finally, plasmid DNA from five bacterial clones (for each H and L chain of each mAb) was purified using the QIAprep Spin Miniprep DNA isolation kit, according to the manufacturer's instructions (QIAGEN). The presence of appropriate size inserts was verified by restriction enzyme digestions. Up to 10 putative positive clones (by region V cloning event) were sequenced with primers T7 and M13Rev, by using the Big Dye Terminator v3.1 (Applied Biosystems), in Genetic Analyzer 3500xl and 3130xl automatic capillary sequencers (Applied Biosystems) according to the Sanger method (23). The consensus nucleotide sequences were generated using the ContigExpress program within the Vector NTI Advance package (Invitrogen) and submitted to GenBank [accession numbers: 4A2_H (MW435573), 4A2_L (MW435574), 1E12_H (MW435575), 1E12_L (MW435576)].

### Sequence and Molecular Structure Data Management and Mining

The general manipulation, analysis, and image production for the aa sequence and Ab molecular structures were conducted with Chimera software (24). Molecular conditioning (e.g., atom protonation and atomic clashes fixes), as a preparatory stage for a mutation task, was implemented with FoldX (25, 26) and MOE (27), which also provides specific modules to perform the alignment of molecular structures and RMSD (root-mean-square deviation of atomic positions) calculus.

The specific calculation and analysis of diverse molecular features of the mAbs, e.g., CDR definition and probability calculations for various kinds of interactions at paratope's residues, were performed with proABC (28) and Paratome (29). The quality of the obtained mAb molecular models was assessed with Molprobity (30).

### Ab Modeling and Ag–Ab Docking Protocol

The overall process for Ag–Ab modeling and docking was conducted as outlined in Supplementary Figure 1. This process involved three main areas: FMDV Ag, mAbs modeling, and an integrative docking and functional scoring stage. First, the molecular structure of the FMDV A24 Cruzeiro ASA was developed with the FoldX software (25) based on the FMDV AgSA at pdb 1EJO (11). In addition, an ensemble of 30 molecules of the Ag (Ag-M1) was obtained with the Pertmint-MOE software (27, 31).

The nucleotide and the deduced aa sequences for the H and L coding regions of the 1E12 and 4A2 mAbs Fab were first obtained as detailed in the *Cloning Foot-and-Mouth Disease Virus mAbs Coding Sequence* section. Next, multiple molecular mAb models were created by four Ab-dedicated modeling software [KOTAI (32), MOE (27), Rosetta-Antibody (33), and ABodyBuilder (34)]. The quality of the emerging molecular models was evaluated by the MolProbity tool (http://molprobity.biochem.duke.edu/) (30), as suggested by the antibody modeling assessments (AMA) initiative (35). The general denomination for these Ab molecules was mAb-M2. Third, *de novo* Ag–Ab candidate interaction topologies were obtained by two-round computational Ag–Ab docking:

**dA**: An initial general docking stage (dA) was conducted with the Haddock software (high ambiguity-driven protein–protein DOCKing) (36), which is an online information-driven flexible docking approach for the modeling of biomolecular complexes. The protocol for dA was implemented as suggested by Trellet et al. with the “guru interface” webserver's client and using the modeled molecules defined above, mAb-M2 and Ag-M1, as input (37).

**dB**: The emerging top two candidate solutions of every (dA) general docking round became the molecular input for the focused docking stage (dB) implemented using the RosettaDock web server (38, 39). The RosettaDock Server performs a local docking search. That is, the algorithm will search a set of conformations near the given starting conformation for the optimal fit between the two Ag–Ab partners. The resulting Ag–Ab interaction topologies became the input for the last scoring stage.

General modeling and mutations at the FMDV AgSA were computationally accomplished by a single-residue replacement task. Specifically, we employed the FoldX software (academic licensed, http://foldxsuite.crg.eu/) through routine scripts extensively described in the software manual. The scripts for the complex interface repairing (pre-process of the pdb files), mutant building, and energy determination were RepairPDB, BuildModel, and AnalyseComplex, respectively (25, 26). The software yields a mutant complex and a mutant-specific wt complex. These complexes were useful to obtain both, the absolute energetic values of an Ag–Ab interaction, as well as the variation between the mutant and the wt-free energy of unfolding at the interaction interface (ΔG).

### Functional Scoring of Ag–Ab Candidate Interaction Complexes

In addition to the software-specific docking scores, the final candidate solutions for near-native Ag–Ab interaction topologies were selected through a functional score. Specifically, we selected the better-scored topologies from a correlation between the two data sets. The antigenic profile A was built with peptide ELISA data for the 13 AgSA peptides (see section Immunoassays). Second, the antigenic profile B was built from *in silico* interaction energy dataset for the same FMDV variant peptides. The profile B for every candidate topology was calculated using the molecular complex as a template. Every scored candidate solution was pre-treated by a multistate modeling (MSM) approach, which implied the generation of a preparative ensemble of 30 molecular structures for every Ag–Ab interaction with PertMin-MOE software (27, 40). Subsequently, every candidate ensemble became the input for the 13 single point mutant modeling. The variation of the interaction energy associated with each mutation (profile B) was calculated using the FoldX program, according to the routines described above. Last, the estimation of the coefficient of determination (correlation) between the *in silico* mutational profile B and the ELISA mutational profile A became the functional score to point out candidate solutions of near-native Ag–Ab interaction complex, the last macromolecular output of our structural modeling pipeline.

## Results

### Molecular Characterization of Novel Ag–Ab Complexes for Foot-and-Mouth Disease Virus of Serotype A

Antibodies are heterodimeric proteins composed of two heavy (H) and two light (L) chains. The entire macromolecule contains 12 immunoglobulin domain repeats and their variable region, VH and VL domains, form the antigen-binding region (ABR) or paratope. The ABR includes six variable loops termed complementarity-determining regions (CDRs) that are spaced by framework regions (FR) (41).

**Figure 1:**
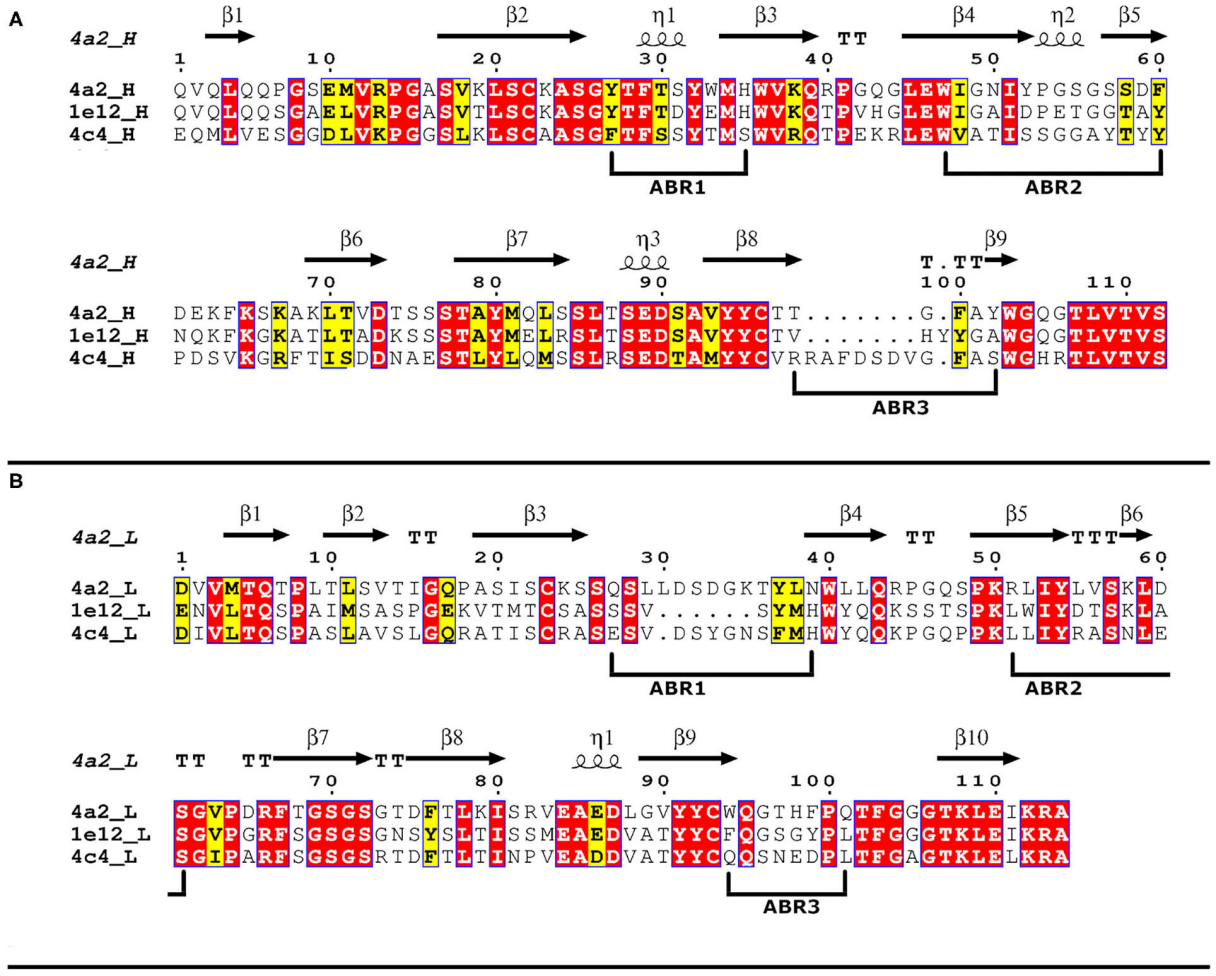
Multiple-sequence alignments of anti-foot-and-mouth disease virus (FMDV) mAb Fv sequences. Amino acid Fv sequences of 4A2, 1E12, and reference 4C4 mAbs. Invariant and conserved residues are highlighted in red and yellow, respectively. Residues are numbered according to the Kabat numbering system and secondary structure elements for variable sequences are indicated above the sequence [spirals, α, and 310(η)-helices; arrows, β-strands; T, turns]. Antigen-binding regions (ABR) are underlined for H **(A)** and L **(B)** chains.

In a previous work, Seki et al. (16) has described several anti-serotype A FMDV mAbs. Here two AgSA-specific mAbs (1E12 and 4A2) (17) were selected for further characterization. First, we amplified the coding sequence of their VH and VL domains (Fv region) by nested RT-PCR, cloned the resulting amplifications, and their nucleotide and their deduced aa sequences were analyzed for (i) the residues and CDRs that shape the paratopes (Figure 1 and Table 1) and (ii) calculating the interaction probability of residues to its cognate epitope (Supplementary Figure 2).

**Table 1:** Classification and number of aa for six ABRs of mAbs, H, and L chains.

	**mAb**								
	**4C4**			**1E12**			**4A2**		
H chain									
ABR #	1	2	3	1	2	3	1	2	3
Clasification	1	O	B	1	2	NB	1	2	short
aa num.	5	17	11	5	17	5	5	17	4
L chain									
ABR #	1	2	3	1	2	3	1	2	3
Clasification	k-5	k-1	k-1	k-1	k-1	k-1	k-4	k-1	k-1
aa num.	15	7	9	10	7	9	16	7	9

*The table displays features for novel, 1E12 and 4A2, and reference 4C4 mAbs. Classification was according to the Kabat–Chothia canonical structures, where “O” (other) refers to H-ABR not belonging to any canonical structure class. For H3 ABRs, the only two canonical structure classes used were B (bulged) and N (non-bulged). For L chains, “k” stands for kappa. The green and yellow boxes correspond to the more relevant CDR in H and L chains, respectively*.

Comparison revealed that 4A2 and 1E12 mAbs displayed smaller paratopes than 4C4 (serotype C mAb reference) and that a lower proportion of the molecular surface of these mAbs was potentially involved in the interaction with the Ag, compared with the reference mAb. Specifically, mAb 4C4 presented a longer H-CDR3 (11 aa) than H-CDR3 of mAb 4A2 (4 aa) and 1E12 (5 aa). The mAb 1E12 also displayed the smallest paratope of the three mAbs because of its shorter L-CDR1 (10 aa) in comparison with 4C4 (15 aa) and 4A2 (16 aa).

The interaction probability data for three different types of molecular contacts of H and L chain paratope residues were consistent with these features. In fact, the prediction of the six loci of greatest probability agreed with the predicted location of the six CDRs (Supplementary Figures 2, 3 for H and L chain, respectively). For L-CDR1, the probability of interactions was higher for mAb 4A2 than for 1E12 (potentially because of a shorter L-CDR1 for the latter).

**Figure 2:**
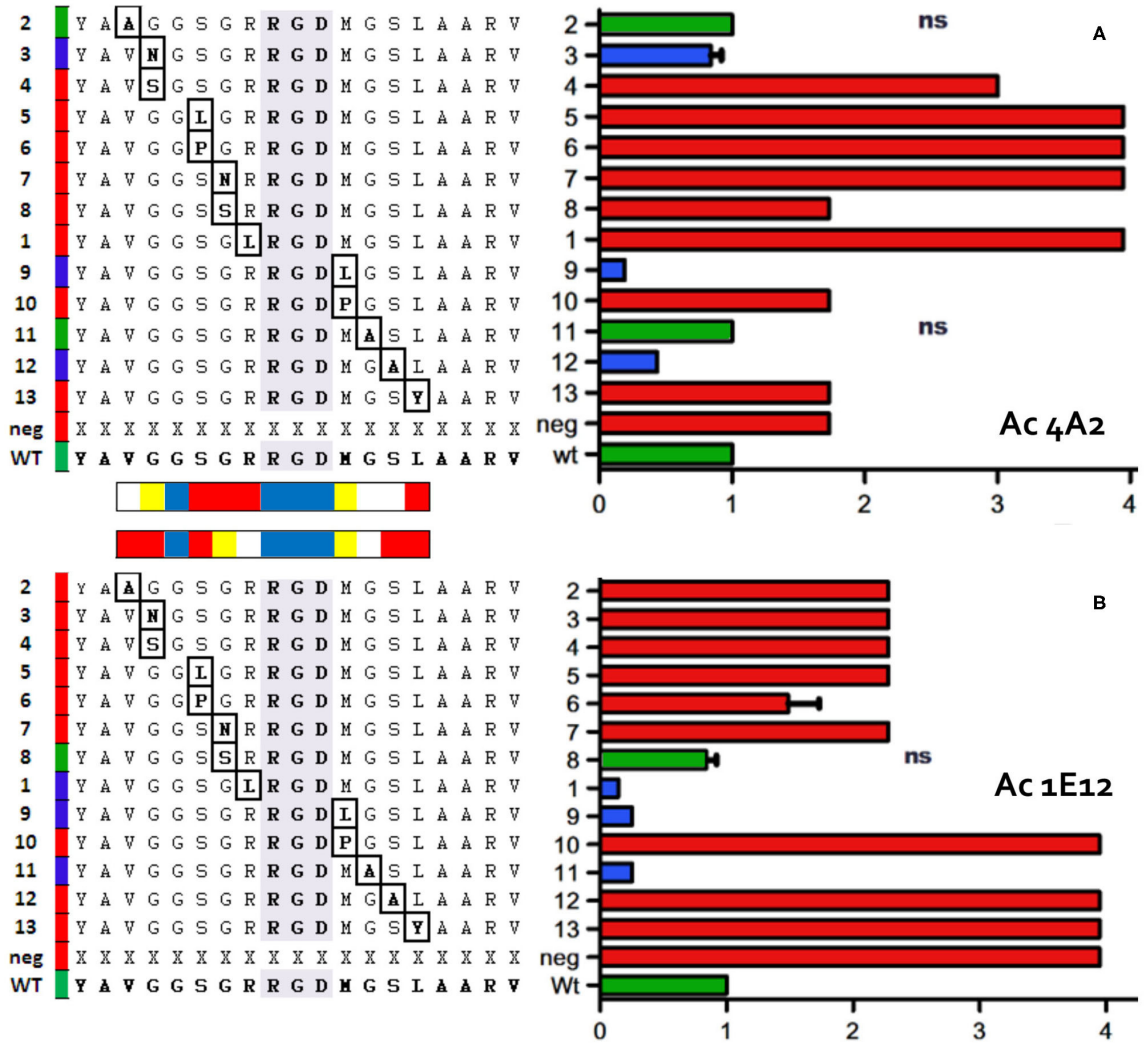
Peptide ELISA mapping of functional epitopes for two anti-FMDV Ab. ELISA results between 15 peptides and mAbs 4A2 or 1E12 are presented in horizontal panels **(A,B)**, respectively. Each panel: Left, the panel displays the specific aa sequences of each peptide; the point mutation is indicated with a box. Right, the panel shows the influence of the point mutations according to the ELISA data; the colors (in the columns) represent IIR50 relative to the wt peptide as follows: in green, the wt peptide and peptides with mutations of IIR50 (relative interaction index 50) equal to wt; in blue, peptides with IIR50 mutations lower than wt (improve interaction) and in red, peptides with IIR50 mutations greater than wt (worsen interaction). The horizontal bar on each peptide matrix summarizes the positions most affected by the mutations in the functional epitope for each mAb, in red and yellow; positions of low and medium tolerance to mutations, respectively, in blue; non-mutated positions and near-native mutations, in white.

We also elaborated a fine map for 1E12- and 4A2-specific epitopes based on previous basic mapping that allocated the recognition site at the AgSA (16). Figure 2 summarizes the findings of several relevant residues (for both epitopes) that overlap at the RGD motif of AgSA with 1E12 epitopes involving more RGD distal residues than mAb 4A2. In detail, the AgSA epitopes were defined through two different peptide-ELISA. To that end, we designed a set of 13 peptides with single point mutations based on the aa usage in several FMDV serotype A strains (Supplementary Table 1).

For 4A2 mAb, we used a peptide-competitive ELISA and observed that mutations downstream and close to the RGD (peptides 4, 5, 6, 7, 8, and 1) were less tolerated (a lower interaction affinity means a poorer competitor peptide). For the same peptides with the 1E12 mAb (sandwich ELISA), we obtained a more RGD-peripheral effect; where peptides 2 and 3 entailed disruptive mutations, however, peptides 8 and 1, with changes closer to the RGD, displayed more tolerated ones. Concerning upstream RGD mutations, both RGD+1 leucine and proline substitutions displayed a similar pattern in both interactions. A proline (peptide 10) was non-tolerated at that short helical fold in the loop GH, although the opposite could be observed with leucine that is more chemically related to the wt aa, methionine (peptide 9). C-terminal mutations demonstrated a similar distal effect, where RGD+4 (peptide 13) was equally disruptive on both interactions, whereas only the 1E12 mAb was sensitive to RGD+3 (peptide 12).

### Development of a Foot-and-Mouth Disease Virus Ag–Ab Tuned Docking Protocol

For further characterization of the sequence and function of mAb-Ag (1E12 and 4A2/FMDV AgSA) interaction complexes, we studied the Ag–Ab molecular structure through a computational modeling approach. However, we first had to define and consolidate an Ag–Ab docking protocol.

To that end, we employed the FMDV Ag–Ab molecular structure of reference [pdb 1EJO (11)] as input. In detail, the exploration of the Ag–Ab interaction topologies was implemented through a two-stage pipeline of docking experiments. First, we evaluated and adjusted a protocol for a general docking stage [software Haddock; (42)] by using individualized Ag and Ab molecules of pdb 1EJO as input (Supplementary Figure 4). The Haddock algorithm can sample diverse docking scenarios, including flexible peptide–protein docking supported by well-documented protocols (37, 43). Then, we obtained solution clusters with acceptable i-rmsd and l-rmsd parameters (<1Å), as defined by the Haddock developers and according to the international initiative for the evaluation of docking protocols [Critical Assessment of Predicted Interactions (CAPRI)] (44).

**Figure 3:**
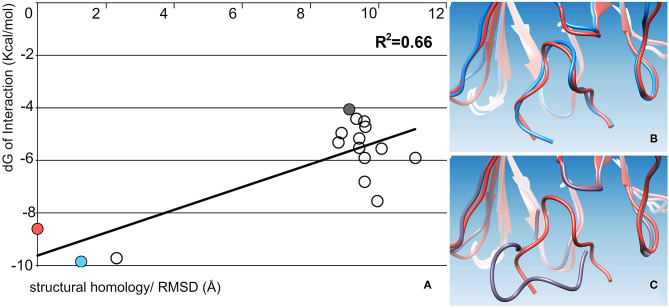
Ag–Ab focused-docking recapitulation for an FMDV interaction complex of reference. The figure displays data for 16 Ag–Ab candidate interaction complexes. **(A)** Regression plot between interface energy score (dG) and RMSD for each Ag–Ab candidate solutions. **(B,C)** exemplifies the structural alignment (of the main chains) between mAbs, revealing the structural distance of FMDV peptide in the wt complex (orange), compared with a top (pale blue), and to a poor (gray) dG scored solution.

Second, we used the emergent top Haddock solutions as an unbiased input for a local docking stage (dB) implemented at the RosettaDock web server (22, 23). That was a focused docking search near the given starting (input) conformation of the optimal fit between the Ag–Ab interaction partners. Some of the best-scored solutions displayed a remarkable structural homology with the native Ag–Ab topology (*R*^2^ of 0.66) for the wt (pdb 1EJO) Ag–Ab complex (Figure 3).

### Molecular Structural Characterization of Novel Ag–Ab Interaction Complexes

After defining the docking protocol (section Development of a foot-and-mouth disease virus Ag–Ab tuned docking protocol), we assessed the Ag–Ab interaction partners (section Molecular characterization of novel Ag–Ab complexes for foot-and-mouth disease virus of serotype A). First, the molecular structure of mAbs 4A2 and 1E12 modeled from its aa sequence by using four different programs (see the section Materials and methods) yielded five and 13 models, respectively. Several of those models displayed molecular quality features comparable with the mAb 4C4 [pdb 1EJO (11); Supplementary Figure 5]. The AgSA of FMDVA24 Cruzeiro was modeled using FoldX protocols (45) and serotype C FMDV AgSA model (at pdb 1EJO) as a template. Then, the Ag was expanded from a single to multiple molecular state representation, which should bring a more dynamic definition of the Ag input for the next “flexible docking” stage (27, 31).

**Figure 4:**
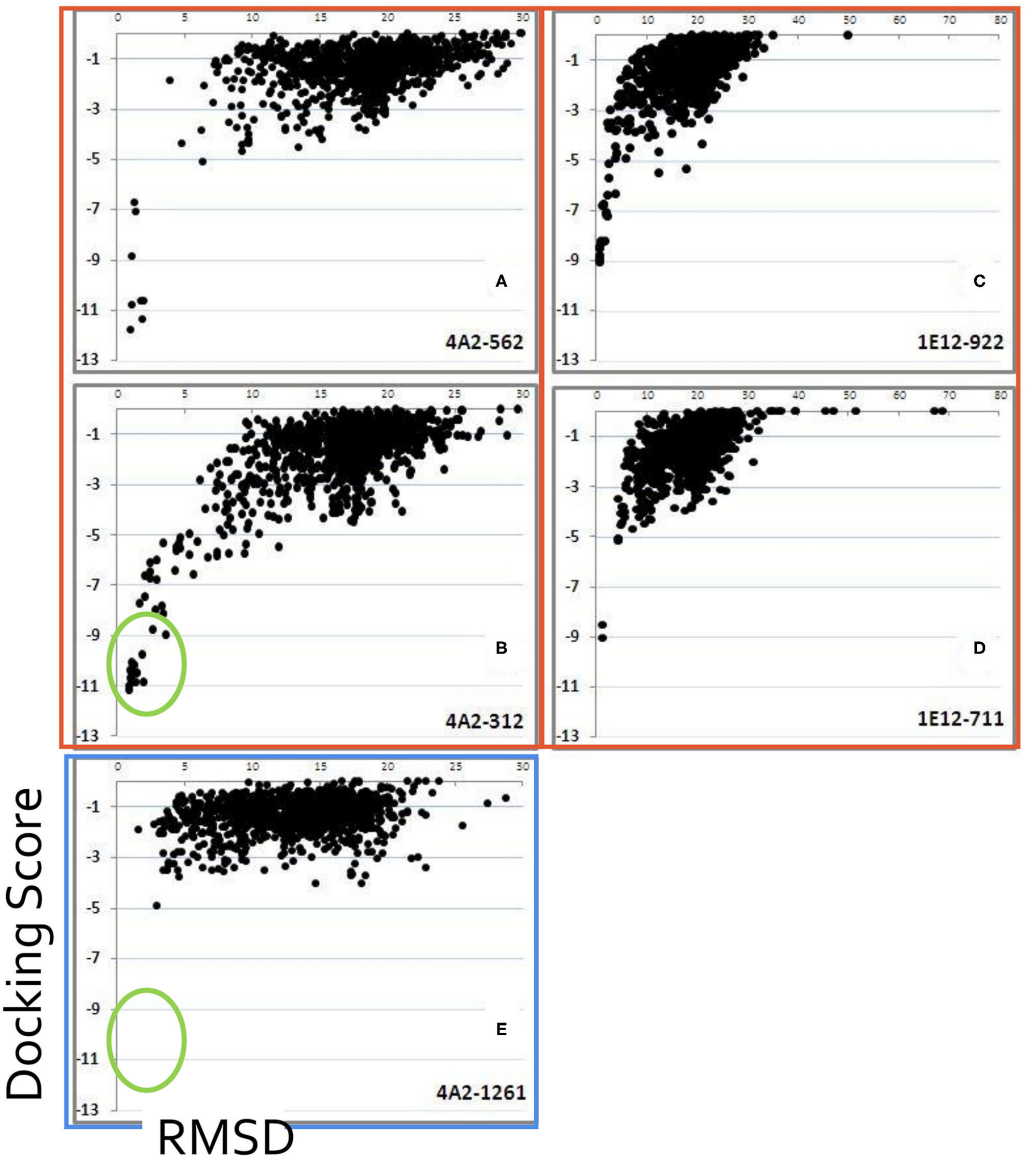
Focused-docking experiments for candidate Ag–Ab complexes. Plots of five different docking experiments using mAb 1E12 or 4A2; dots composed by dG values (Docking score) vs. structural homology values (RMSD, on the horizontal axis). A characteristic pattern called energy funnel **(A–D)**, except for **(E)**, which was a negative control) emerges at near-native docking solutions. Green circles at **(B,E)** denotes the presence or absence of the energy funnel pattern.

The sampling of the interaction space for both FMDV AgSA and mAb models using the previous tuned docking protocol (Supplementary Figure 1) retrieved five and eight candidate Ag–Ab docking solutions for mAb 4A2 and 1E12 complexes, respectively. All those topologies displayed the characteristic “energy funnel” pattern in a dG score vs. an RMSD plot (Figure 4), a feature associated with candidate solutions sampling near the native interaction state (39, 46). Furthermore, the implementation of a functional score biased the results in favor of those candidate Ag–Ab complexes with best mutational fitness between its mutational antigenic profile and a peptide ELISA profile (see the section Molecular characterization of novel Ag–Ab complexes for foot-and-mouth disease virus of serotype A).

**Figure 5:**
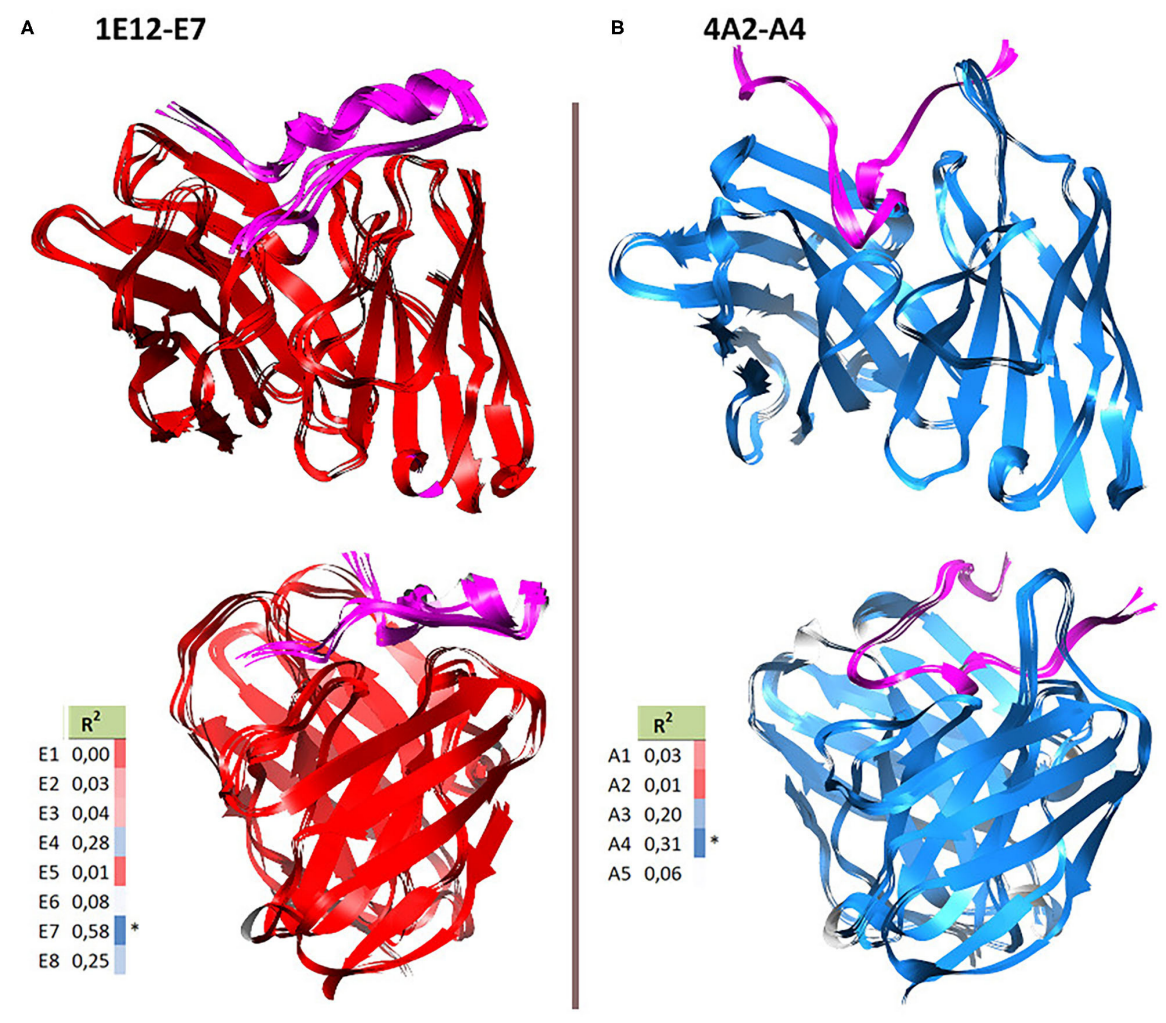
Top docking solutions emerging from the integral modeling protocol. Two perspectives of the interaction topologies between the FMDV AgSA peptide (pink) and mAbs 1E12 (red) and 4A2 (blue) are shown in vertical panels **(A,B)**, respectively. Note that the peptide has a flatter interaction with 1E12 mAb, unlike the interaction with 4A2, where the peptide would be more embedded in the paratope.

**Figure 6:**
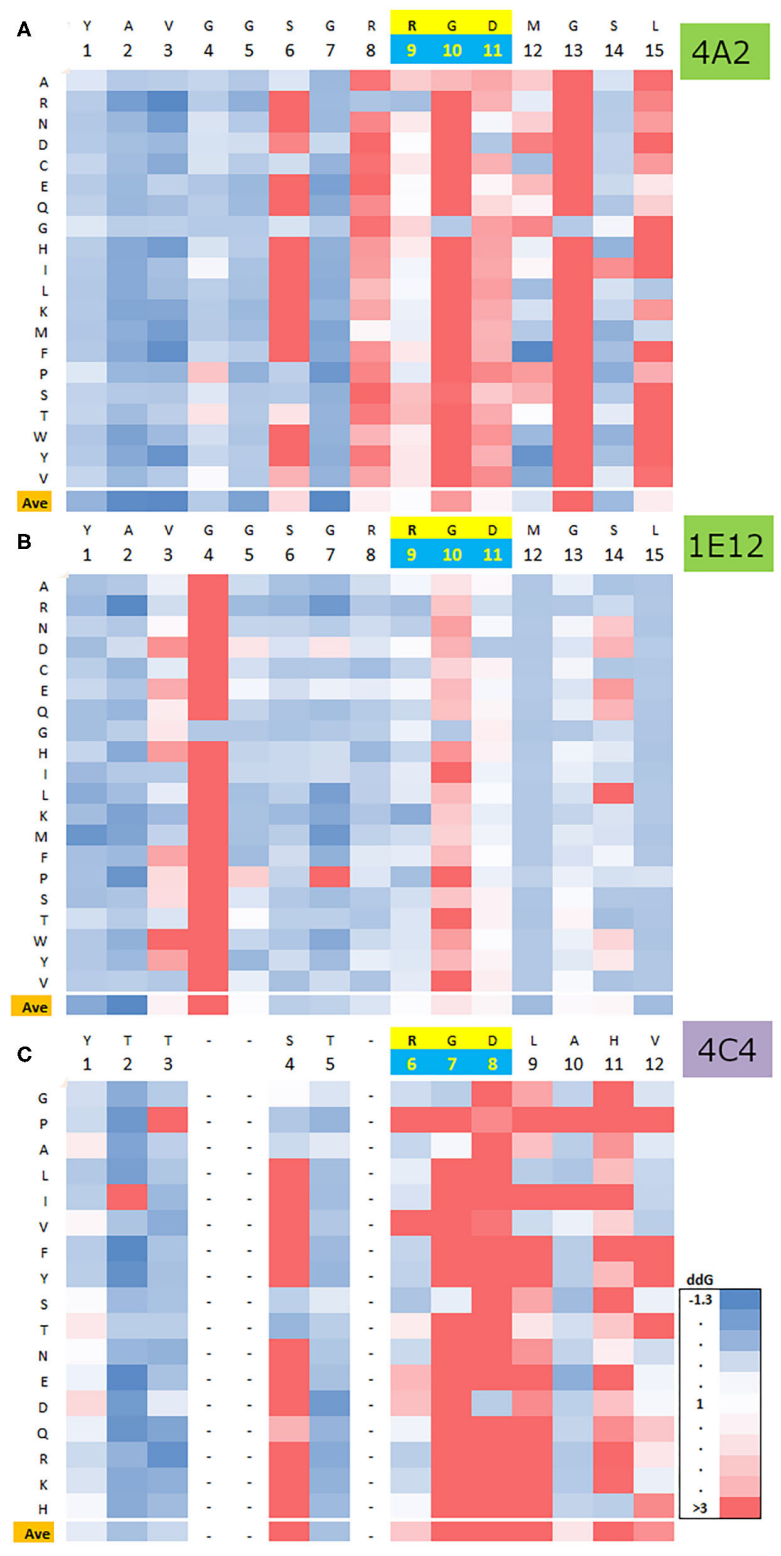
AgSA mutational profile for three FMDV Ag–Ab complexes. Computational mining and comparison of the influence of single point mutations at the FMDV Ag partner for two Ag–Ab complexes and one Ag–Ab complex of reference. Horizontally, each panel contains the native FMDV AgSA sequence (see the conserved RGD motif in yellow) and, vertically, the energetic effect (dG) of single point aa mutations. **(A,B)** correspond to mAbs 4A2 and 1E12 interaction complexes, respectively, and **(C)** displays reference 4C4 mAb (pdb 1EJO) interaction data. Color variation from blue to red implies a worsening of the interaction energy and a less tolerated mutation at a given Ag–Ab. See the similarities between **(A,C)** involving IgG Abs and a different mutational profile for the IgM Ab **(B)**.

For further characterization of top Ag–Ab solutions (Figure 5), we modeled and calculated the effect of every single point mutation per each AgSA residue. That computational exploration of the Ag Tolerated Sequence Space (TSS) was previously demonstrated as feasible by our group (45). Here we obtained three TSS, one for the AgSA-4c4 mAb complex (serotype C reference) and the other two for the novel anti-serotype A mAbs complexes. Figure 6 illustrates the existence of similar TSS patterns between the “IgG interactions,” in opposition to the IgM 1E12-AgSA complex that displayed a more mutation-permissive pattern.

## Discussion

Antibody-mediated neutralization is one of the major host mechanisms to decrease and resolve the FMDV infection (47, 48); hence, understanding the Ag–Ab molecular interaction is an essential condition for the development of better FMDV biotechnology tools for disease control (49, 50).

Besides, recent SARS-CoV-2 research has demonstrated a feasible synergy between Ag–Ab molecular structure knowledge and “big data” information from full Ab repertoire analysis (51–53). For FMD, the immunogenomics and polyclonal antibody response have been initially dissected in cattle and African buffaloes (8, 9, 54). With a different approach, we explored and computationally interrogated the sequence space of Ag–Ab interactions at FMDV AgSA of serotype C, and we completed successfully an *in silico* study between two mAbs and more than 200 single point AgSA mutants (45).

Here, we relied on structural data for reference FMDV Ag–Ab complex (pdb 1EJO) to implement a full *in silico* molecular modeling pipeline for the FMDV AgSA–Ab interactions. The adjusted protocol was applied to two novel Ag–Ab interactions for another relevant South American FMDV of serotype A (A24 Cruzeiro). In addition, we added a functional-based score to assist the computational exploration of near-native Ag–Ab candidate solutions. A similar data-assisted docking approach has been defined as applicable for docking-algorithm-dedicated research groups (43, 55). Although molecular docking techniques are prone to diverse errors in non-guided experiments (56, 57), here we detected a better predictive performance at the FMDV AgSA scenario that could be associated with the intrinsic simple folding at the RGD turn of the GH loop (58), which means more computational-tractable linear epitopes.

Novel FMDV mAbs Fab sequences were cloned, and their epitopes were characterized by two different ELISAs. The results revealed that the 1E12-specific epitope involved more RGD-distal residues than the mAb 4A2-specific epitope. In addition, both 1E12 and 4A2 mAbs presented smaller paratopes than the reference FMDV 4C4 mAb. However, 4A2 displayed a slight increase in charged residue usage, which could be associated with its characteristic IgG isotype form. Indeed, some researchers have previously reported a net gain of positive charges in IgG in comparison with IgM paratopes (59). Also, mAb 4A2, which is more similar to mAbs 4C4 and SD6 (all of them IgG), featured a long CDR-L1 associated with Abs with greater affinity for protuberant antigens such as the GH loop of FMDV (5). The relevance of the aa positions near the RGD that shapes a linear epitope is undeniable (58). However, in this work, we demonstrated that different residues could have a very distinctive influence on the interaction of the studied Ab–Ag complexes.

Another distinguishing characteristic between the IgM and the IgG Ab is that the molecular modeled structure for mAbs 1E12 and 4A2 displayed a different topology at their paratopes. Specifically, top solutions for mAb 4A2 showed a concave paratope similar to IgG Ab models described for FMDV of serotype C [pdb 1EJO; (11)]. This feature differed from the 1E12 paratope (an Ab of IgM isotype), whose top-ranked molecular model presented a flatter topology of interaction for the same Ag, supporting the differential interaction data obtained by Seki et al. (16). In their work, both were neutralizing Abs, although 1E12 displayed a greater “poly-specificity,” as it was positively recognized by more AgSA mutations in FDMV isolates (16). In recent years, there has been a growing interest regarding the relevance of IgM to the FMDV host humoral response (60, 61). Experimental evidence indicates that virus neutralization in vaccinated or infected cattle could be greatly mediated by IgM subtype antibodies by means of thymus-independent humoral immune responses (62). In addition, we obtained more similar TSS patterns between the “IgG interactions” (Figures 6A,C) than the IgM 1E12–AgSA complex, which displayed a more mutation-permissive pattern. This contrasting feature could be associated with the above-referred differences at the paratope topology and the AgSA peptide orientation and insertion, concerning the Ab's grooves (Figure 5). This could be observed at the TTS differences for the four residues downstream of the RGD motif that build a short helical fold.

Our results suggest the existence of a typical structural diversity of the Ag–Ab modes of interaction at the FMDV AgSA, in contrast with a previous report for mAbs 4C4 and SD6 that showed a greater structural and functional homology at their interaction with a homolog AgSA ligand (FMDV of serotype C). Verdaguer et al. (11) argued the putative existence of a structural bias at the Ab repertoire for the interactions. An impediment in the host humoral response is associated with the presence of the viral RGD motif, which would be acting as a self-antigen (a motif found at several host processes and proteins). However, this contrasting finding could be also attributable to the different isotype nature of the Abs used in our study. For example, a strikingly high aa homology was found between mAb 4A2 with two IgG mAbs (F24G3 and F24G1), also active against A24 Cruzeiro AgSA, produced by another research group (63). In a multiple sequence alignment, the mAbs not only presented a great H chain homology but also contained identical aa sequences at their CDR3-H (Supplementary Figure 6) (64). These speculations coming from few examples should be further explored by detailed *in vivo* characterization.

In summary, we generated novel information related to the structural diversity of interactions between epitopes of the FMDV antigenic site and two FMDV Abs of IgM and IgG isotypes. The present work provides evidence about the different characteristics of both isotypes but also points out the similarities among the induced IgG ab structures. This information could be of great biotechnological relevance for the development of new antigens and vaccines with improved cross-protection features. These predictive models, combined with NGS repertoire antibody information, offer a promising tool for the FMDV epidemiology and vaccinology applications (64).

## Data Availability Statement

The datasets presented in this study can be found in online repositories. The names of the repository/repositories and accession number(s) can be found in the article/Supplementary Material.

## Author Contributions

RM and GK designed the study. RM, CS, and GK collected the data and performed the subsequent analyses. RM prepared the manuscript. RM, CS, NM, and GK edited the manuscript. All authors have read and approved the manuscript.

## Conflict of Interest

The authors declare that the research was conducted in the absence of any commercial or financial relationships that could be construed as a potential conflict of interest.

## Supplementary Material

Supplementary Figure 1 Ag-Ab modeling pipeline. The black outlined rectangles define input points of sequences to the protocol whereas, red, green, blue, and yellow colors refer to modeling stages for the Ag, mAbs, Ag-Ab complexes, and candidate mutational scoring, respectively.

Supplementary Figure 2 H chain-specific mapping of Ag-Ab contact probability. Contact probability at H chain residues of different Abs and FMDV ASA. Data are displayed as a probability contact over the H chains residues of the reference mAb 4C4 (green), and mAbs 4A2 (blue) and 1E12 (red). The contacts are presented from left to right as follows: A, non-bonded interactions; B, Hydrogen bridge interactions (polar) and C, hydrophobic interactions (apolar).

Supplementary Figure 3 L chain-specific mapping of Ag-Ab interaction probability. Non-bonded (NB) interaction probability data per aa residues of the L chains are depicted. The reference 4C4 mAb (green) plot is superposed to 4A2 mAb (blue; A) and 1E12 mAb (red; B) plots.

Supplementary Figure 4 Graphic output from the result page of the HADDOCK web server relative to three different molecular docking experiments. The panels display variable docking clusters obtained from 3 different docking inputs of the same FMDV peptide Ag. Single-state Ag input (A) and two different multiple-state Ag input molecular ensembles obtained after 10 (B) or 50 (C) x,y,z coordinates subtle perturbations cycles. The graphics are based on water-refined models generated by HADDOCK. The clusters (indicated in color in the graphs) are calculated based on the interface-ligand RMSDs assessed by HADDOCK, with the interface defined automatically according to all observed contacts. The various FCC, i-RMSD, and l-RMSD structural analyses are made with the best HADDOCK model (the one with the lowest HADDOCK score). Interface-RMSD (i-RMSD) calculated on the backbone (CA, C, N, O, P) atoms of all residues involved in intermolecular contact using a 10Å cutoff. Ligand-RMSD (l-RMSD) calculated on the backbone atoms (CA, C, N, O, P) of all (N > 1) molecules after fitting on the backbone atoms of the first (N = 1) molecule. The average values are calculated on the best 4 structures of each cluster (based on the HADDOCK score). Cluster averages and standard deviations are indicated by colored dots with associated error bars.

Supplementary Figure 5 Quality scores for Ab molecular models. The scoring, according to MolProbity, includes three metrics (cells in blue) with values that improve according to a scale of colors from red to green. The tables reflect data from 3 models for each of the mAbs 4A2 and 1E12, as well as data for the reference mAb 4C4.

Supplementary Figure 6 Multiple sequence alignment for anti-A24 cruzeiro FMDV mAbs. H-chain aa. sequence alignment between 4A2 mAb (seq. A41-HL) and 2 mAbs developed (F24G3 and G1) against the same FMDV strain. The red boxes indicate CDR1 regions.

Supplementary Table 1 List of mutant peptides. Mutations and RGD motifs are bold-highlighted. (*)Leu aa usage to contrast the Arg aa prevalence. (**)Pro and Tyr aa usage as chemical structure modifiers for the RGD +1 +4 short helix span.

## References

[1] Knight-Jones TJ Robinson L Charleston B Rodriguez LL Gay CG Sumption KJ. Global foot-and-mouth disease research update and gap analysis: 1 - overview of global status and research needs. Transbound Emerg Dis. (2016) 63(Suppl 1):3–13. 10.1111/tbed.12528 27320162

[2] Feigelstock DA Mateu MG Valero ML Andreu D Domingo E Palma EL. Emerging foot-and-mouth disease virus variants with antigenically critical amino acid substitutions predicted by model studies using reference viruses. Vaccine. (1996) 14:97–102. 10.1016/0264-410X(95)00180-9 8852403

[3] Mateu MG Martinez MA Capucci L Andreu D Giralt E Sobrino F. A single amino acid substitution affects multiple overlapping epitopes in the major antigenic site of foot-and-mouth disease virus of serotype C. J Gen Virol. (1990) 71(Pt 3):629–37. 10.1099/0022-1317-71-3-629 1690261

[4] Lannes N Python S Summerfield A. Interplay of foot-and-mouth disease virus, antibodies and plasmacytoid dendritic cells: virus opsonization under non-neutralizing conditions results in enhanced interferon-alpha responses. Vet Res. (2012) 43:64. 10.1186/1297-9716-43-64 22934974 PMC3479418

[5] Finlay WJ Almagro JC. Natural and man-made V-gene repertoires for antibody discovery. Front Immunol. (2012) 3:342. 10.3389/fimmu.2012.00342 23162556 PMC3498902

[6] Shimmon G Kotecha A Ren J Asfor AS Newman J Berryman S. Generation and characterisation of recombinant FMDV antibodies: applications for advancing diagnostic and laboratory assays. PLoS ONE. (2018) 13:e0201853. 10.1371/journal.pone.0201853 30114227 PMC6095514

[7] Grant CFJ. Investigating primary and secondary B cell responses in cattle after immunisation with existing and novel vaccines [Master's thesis]. University of Oxford (2013).

[8] Philp RL. The Polyclonal Antibody Response to FMDV in Cattle and African buffalo [degree of Doctor of Philosophy]. University of Glasgow (2018).

[9] Hammond J. Dissecting polyclonal responses. In: WRLFMD 60th Anniversary Symposium. UK2018 (2018).

[10] Cao Y Li K Wang S Fu Y Sun P Li P. Implication of broadly neutralizing bovine monoclonal antibodies in the development of an enzyme-linked immunosorbent assay for detecting neutralizing antibodies against foot-and-mouth disease virus serotype. J Clin Microbiol. (2019) 57:1–9. 10.1128/JCM.01030-19 31578261 PMC6879294

[11] Verdaguer N Sevilla N Valero ML Stuart D Brocchi E Andreu D. A similar pattern of interaction for different antibodies with a major antigenic site of foot-and-mouth disease virus: implications for intratypic antigenic variation. J Virol. (1998) 72:739–48. 10.1128/JVI.72.1.739-748.1998 9420281 PMC109430

[12] Ochoa WF Kalko SG Mateu MG Gomes P Andreu D Domingo E. A multiply substituted G-H loop from foot-and-mouth disease virus in complex with a neutralizing antibody: a role for water molecules. J Gen Virol. (2000) 81(Pt 6):1495–505. 10.1099/0022-1317-81-6-1495 10811933

[13] Hewat EA Verdaguer N Fita I Blakemore W Brookes S King A. Structure of the complex of an Fab fragment of a neutralizing antibody with foot-and-mouth disease virus: positioning of a highly mobile antigenic loop. EMBO J. (1997) 16:1492–500. 10.1093/emboj/16.7.1492 9130694 PMC1169753

[14] Marks C Deane CM. How repertoire data are changing antibody science. J Biol Chem. (2020) 295:9823–37. 10.1074/jbc.REV120.010181 32409582 PMC7380193

[15] Sobrino F Davila M Ortin J Domingo E. Multiple genetic variants arise in the course of replication of foot-and-mouth disease virus in cell culture. Virology. (1983) 128:310–8. 10.1016/0042-6822(83)90258-1 6310859

[16] Seki C Robiolo B Periolo O Iglesias M D'Antuono A Maradei E. Rapid methodology for antigenic profiling of FMDV field strains and for the control of identity, purity and viral integrity in commercial virus vaccines using monoclonal antibodies. Vet Microbiol. (2009) 133:239–51. 10.1016/j.vetmic.2008.07.011 18774662

[17] Mahapatra M Seki C Upadhyaya S Barnett PV La Torre J Paton DJ. Characterisation and epitope mapping of neutralising monoclonal antibodies to A24 Cruzeiro strain of FMDV. Vet Microbiol. (2011) 149:242–7. 10.1016/j.vetmic.2010.11.003 21144677

[18] Hornbeck PV. Enzyme-linked immunosorbent assays. Curr Protocols Immunol. (2015) 110:21–3. 10.1002/0471142735.im0201s110 26237010

[19] Carter JM. Production of anti-peptide antisera. Curr Protocols Immunol. (2003) Chapter 9:Unit 9 3. 10.1002/0471142735.im0903s55 18432916

[20] Morrison SL. Cloning, expression, and modification of antibody V regions. Curr Protocols Immunol. (2002) 47:2.12.1–2.12.17. 10.1002/0471142735.im0212s47 18432877

[21] Kabat EA National Institutes of H Columbia U. Sequences of Proteins of Immunological Interest. Bethesda, MD: U.S. Dept. of Health and Human Services, Public Health Service, National Institutes of Health (1991).

[22] Coloma MJ Larrick JW Ayala M Gavilondo-Cowley JV. Primer design for the cloning of immunoglobulin heavy-chain leader-variable regions from mouse hybridoma cells using the PCR. BioTechniques. (1991) 11:152–6. 1931008

[23] Sanger F Nicklen S Coulson AR. DNA sequencing with chain-terminating inhibitors. Proc Natl Acad Sci USA. (1977) 74:5463–7. 10.1073/pnas.74.12.5463 271968 PMC431765

[24] Pettersen EF Goddard TD Huang CC Couch GS Greenblatt DM Meng EC. UCSF Chimera–a visualization system for exploratory research and analysis. J Computat Chem. (2004) 25:1605–12. 10.1002/jcc.20084 15264254

[25] Schymkowitz J Borg J Stricher F Nys R Rousseau F Serrano L. The FoldX web server: an online force field. Nucleic Acids Res. (2005) 33:W382–8. 10.1093/nar/gki387 15980494 PMC1160148

[26] Kiel C Wohlgemuth S Rousseau F Schymkowitz J Ferkinghoff-Borg J Wittinghofer F. Recognizing and defining true Ras binding domains II: *in silico* prediction based on homology modelling and energy calculations. J Mol Biol. (2005) 348:759–75. 10.1016/j.jmb.2005.02.046 15826669

[27] ULC CCG. Molecular Operating Environment (MOE) 2013.08. 2017.

[28] Olimpieri PP Chailyan A Tramontano A Marcatili P. Prediction of site-specific interactions in antibody-antigen complexes: the proABC method and server. Bioinformatics. (2013) 29:2285–91. 10.1093/bioinformatics/btt369 23803466 PMC3753563

[29] Kunik V Ashkenazi S Ofran Y. Paratome: an online tool for systematic identification of antigen-binding regions in antibodies based on sequence or structure. Nucleic Acids Res. (2012) 40:W521–4. 10.1093/nar/gks480 22675071 PMC3394289

[30] Chen VB Arendall WB Headd JJ Keedy DA Immormino RM Kapral GJ. MolProbity: all-atom structure validation for macromolecular crystallography. Acta Crystallogr Biol Crystallogr. (2010) 66(Pt 1):12–21. 10.1107/S0907444909042073 20057044 PMC2803126

[31] Davey JA Chica RA. Improving the accuracy of protein stability predictions with multistate design using a variety of backbone ensembles. Proteins. (2014) 82:771–84. 10.1002/prot.24457 24174277

[32] Yamashita K Ikeda K Amada K Liang S Tsuchiya Y Nakamura H. Kotai antibody builder: automated high-resolution structural modeling of antibodies. Bioinformatics. (2014) 30:3279–80. 10.1093/bioinformatics/btu510 25064566

[33] Sircar A Kim ET Gray JJ. RosettaAntibody: antibody variable region homology modeling server. Nucleic Acids Res. (2009) 37:W474–9. 10.1093/nar/gkp387 19458157 PMC2703951

[34] Leem J Dunbar J Georges G Shi J Deane CM. A body builder: automated antibody structure prediction with data-driven accuracy estimation. mAbs. (2016) 8:1259–68. 10.1080/19420862.2016.1205773 27392298 PMC5058620

[35] Almagro JC Beavers MP Hernandez-Guzman F Maier J Shaulsky J Butenhof K. Antibody modeling assessment. Proteins. (2011) 79:3050–66. 10.1002/prot.23130 21935986

[36] de Vries SJ van Dijk AD Krzeminski M van Dijk M Thureau A Hsu V. HADDOCK versus HADDOCK: new features and performance of HADDOCK2.0 on the CAPRI targets. Proteins. (2007) 69:726–33. 10.1002/prot.21723 17803234

[37] Trellet M Melquiond AS Bonvin AM. A unified conformational selection and induced fit approach to protein-peptide docking. PLoS ONE. (2013) 8:e58769. 10.1371/journal.pone.0058769 23516555 PMC3596317

[38] Schueler-Furman O Wang C Baker D. Progress in protein-protein docking: atomic resolution predictions in the CAPRI experiment using RosettaDock with an improved treatment of side-chain flexibility. Proteins. (2005) 60:187–94. 10.1002/prot.20556 15981249

[39] Lyskov S Gray JJ. The RosettaDock server for local protein-protein docking. Nucleic Acids Res. (2008) 36:W233–8. 10.1093/nar/gkn216 18442991 PMC2447798

[40] Davey JA Chica RA. Multistate Computational Protein Design: Theories, Methods, and Applications Ottawa. University of Ottawa (2016).

[41] Sela-Culang I Kunik V Ofran Y. The structural basis of antibody-antigen recognition. Front Immunol. (2013) 4:302. 10.3389/fimmu.2013.00302 24115948 PMC3792396

[42] van Zundert GCP Rodrigues J Trellet M Schmitz C Kastritis PL Karaca E. The HADDOCK2.2 web server: user-friendly integrative modeling of biomolecular complexes. J Mol Biol. (2016) 428:720–5. 10.1016/j.jmb.2015.09.014 26410586

[43] Trellet M Melquiond AS Bonvin AM. Information-driven modeling of protein-peptide complexes. Methods Mol Biol. (2015) 1268:221–39. 10.1007/978-1-4939-2285-7_10 25555727

[44] Janin J. Protein-protein docking tested in blind predictions: the CAPRI experiment. Mol BioSyst. (2010) 6:2351–62. 10.1039/c005060c 20725658

[45] Marrero R Limardo RR Carrillo E Konig GA Turjanski AG. A computational study of the interaction of the foot and mouth disease virus VP1 with monoclonal antibodies. J Immunol Methods. (2015) 425:51–7. 10.1016/j.jim.2015.06.008 26093030

[46] London N Schueler-Furman O. Funnel hunting in a rough terrain: learning and discriminating native energy funnels. Structure. (2008) 16:269–79. 10.1016/j.str.2007.11.013 18275818

[47] Doel TR. Natural and vaccine induced immunity to FMD. Curr Top Microbiol Immunol. (2005) 288:103–31. 10.1007/3-540-27109-0_5 15648176

[48] Mignaqui AC Ruiz V Durocher Y Wigdorovitz A. Advances in novel vaccines for foot and mouth disease: focus on recombinant empty capsids. Crit Rev Biotechnol. (2019) 39:306–20. 10.1080/07388551.2018.1554619 30654663

[49] Belsham GJ Kristensen T Jackson T. Foot-and-mouth disease virus: prospects for using knowledge of virus biology to improve control of this continuing global threat. Virus Res. (2020) 281:197909. 10.1016/j.virusres.2020.197909 32126297

[50] Shahriari A Habibi-Pirkoohi M. Developing vaccines against foot-and-mouth disease: a biotechnological approach. Arch Razi Institute. (2018) 73:1–10. 30256033 10.22092/ARI.2018.114054

[51] Nielsen SCA Yang F Jackson KJL Hoh RA Roltgen K Jean GH. Human B cell clonal expansion and convergent antibody responses to SARS-CoV-2. Cell Host Microbe. (2020) 28:516–25.e5. 10.1016/j.chom.2020.09.002 32941787 PMC7470783

[52] Meng W Rosenfeld AM Luning Prak ET. Mining the antibody repertoire for solutions to SARS-CoV-2. Cell Host Microbe. (2020) 28:499–501. 10.1016/j.chom.2020.09.010 33031765 PMC7539899

[53] Ravichandran S Coyle EM Klenow L Tang J Grubbs G Liu S. Antibody signature induced by SARS-CoV-2 spike protein immunogens in rabbits. Sci Translat Med. (2020) 12:eabc3539. 10.1126/scitranslmed.abc3539 32513867 PMC7286538

[54] Mahapatra M Parida S. Foot and mouth disease vaccine strain selection: current approaches and future perspectives. Exp Rev Vaccines. (2018) 17:577–91. 10.1080/14760584.2018.1492378 29950121

[55] Kurcinski M Blaszczyk M Ciemny MP Kolinski A Kmiecik S. A protocol for CABS-dock protein-peptide docking driven by side-chain contact information. Biomed Eng Online. (2017) 16(Suppl 1):73. 10.1186/s12938-017-0363-6 28830545 PMC5568604

[56] Rodrigues JP Melquiond AS Karaca E Trellet M van Dijk M van Zundert GC. Defining the limits of homology modeling in information-driven protein docking. Proteins. (2013) 81:2119–28. 10.1002/prot.24382 23913867

[57] de Vries SJ Melquiond AS Kastritis PL Karaca E Bordogna A van Dijk M. Strengths and weaknesses of data-driven docking in critical assessment of prediction of interactions. Proteins. (2010) 78:3242–9. 10.1002/prot.22814 20718048

[58] Mateu MG Verdaguer N. Functional and structural aspects of the interaction of Foot-and-Mouth Disease Virus with antibodies. In: Sobrino F Domingo E, editors. Foot-and-Mouth Disease Virus: Current Perspective Horizon Bioscience. (2004). p. 223–60. 10.1201/9780429125614-9

[59] DeKosky BJ Lungu OI Park D Johnson EL Charab W Chrysostomou C. Large-scale sequence and structural comparisons of human naive and antigen-experienced antibody repertoires. Proc Natl Acad Sci USA. (2016) 113:E2636–45. 10.1073/pnas.1525510113 27114511 PMC4868480

[60] Pega J Bucafusco D Di Giacomo S Schammas JM Malacari D Capozzo AV. Early adaptive immune responses in the respiratory tract of foot-and-mouth disease virus-infected cattle. J Virol. (2013) 87:2489–95. 10.1128/JVI.02879-12 23255811 PMC3571376

[61] Pega J Di Giacomo S Bucafusco D Schammas JM Malacari D Barrionuevo F. Systemic foot-and-mouth disease vaccination in cattle promotes specific antibody-secreting cells at the respiratory tract and triggers local anamnestic responses upon aerosol infection. J Virol. (2015) 89:9581–90. 10.1128/JVI.01082-15 26157128 PMC4542361

[62] Carr BV Lefevre EA Windsor MA Inghese C Gubbins S Prentice H. CD4+ T-cell responses to foot-and-mouth disease virus in vaccinated cattle. J Gen Virol. (2013) 94(Pt 1):97–107. 10.1099/vir.0.045732-0 23034593 PMC3542717

[63] Yuan X Gubbins MJ Berry JD. A simple and rapid protocol for the sequence determination of functional kappa light chain cDNAs from aberrant-chain-positive murine hybridomas. J Immunol Methods. (2004) 294:199–207. 10.1016/j.jim.2004.09.001 15604028

[64] Galson JD Pollard AJ Truck J Kelly DF. Studying the antibody repertoire after vaccination: practical applications. Trends Immunol. (2014) 35:319–31. 10.1016/j.it.2014.04.005 24856924

