# Supplementary figures and images for: Functional and in silico Characterization of Neutralizing Interactions Between Antibodies and the Foot-and-Mouth Disease Virus Immunodominant Antigenic Site
Source: Front Vet Sci. 2021 May 7;8:554383. doi: 10.3389/fvets.2021.554383 (PMC8137985; doi:10.3389/fvets.2021.554383)

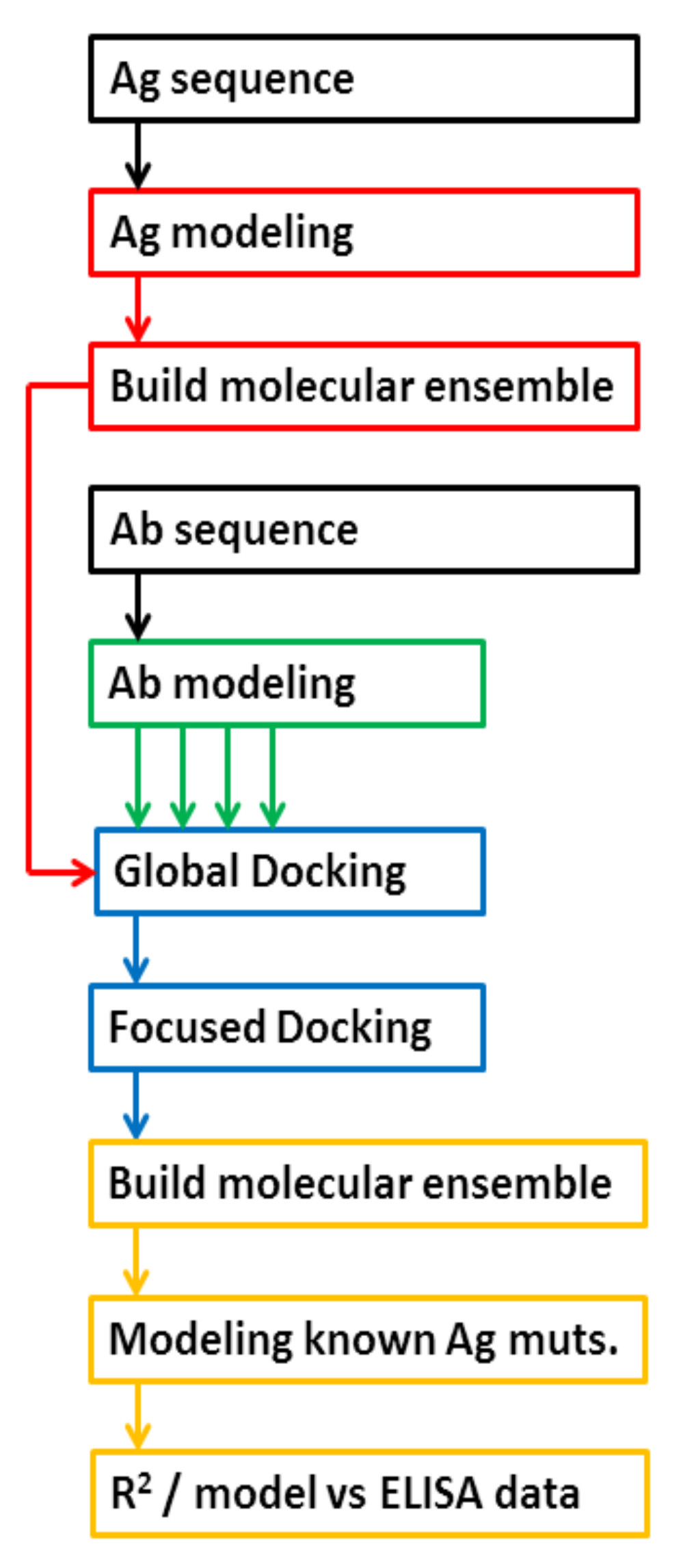

Supplement: Supplementary Figure 1 — Ag-Ab modeling pipeline. The black outlined rectangles define input points of sequences to the protocol whereas, red, green, blue, and yellow colors refer to modeling stages for the Ag, mAbs, Ag-Ab complexes, and candidate mutational scoring, respectively. [file Image_1.JPEG]

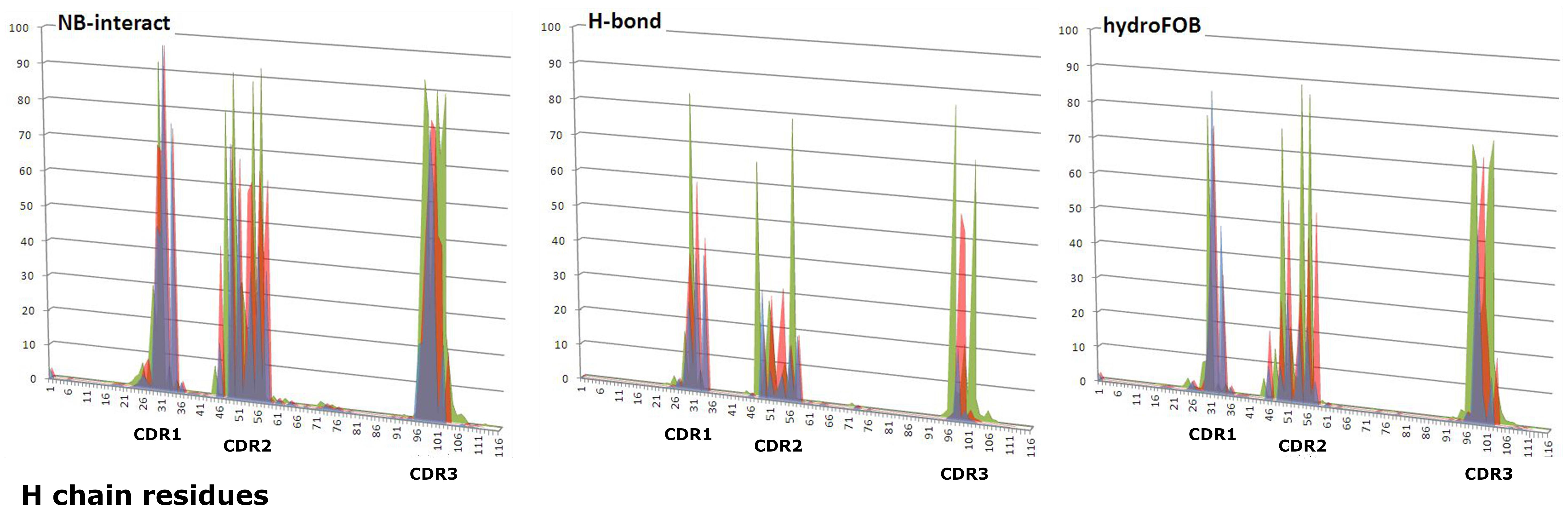

Supplement: Supplementary Figure 2 — H chain-specific mapping of Ag-Ab contact probability. Contact probability at H chain residues of different Abs and FMDV ASA. Data are displayed as a probability contact over the H chains residues of the reference mAb 4C4 (green), and mAbs 4A2 (blue) and 1E12 (red). The contacts are presented from left to right as follows: A, non-bonded interactions; B, Hydrogen bridge interactions (polar) and C, hydrophobic interactions (apolar). [file Image_2.JPEG]

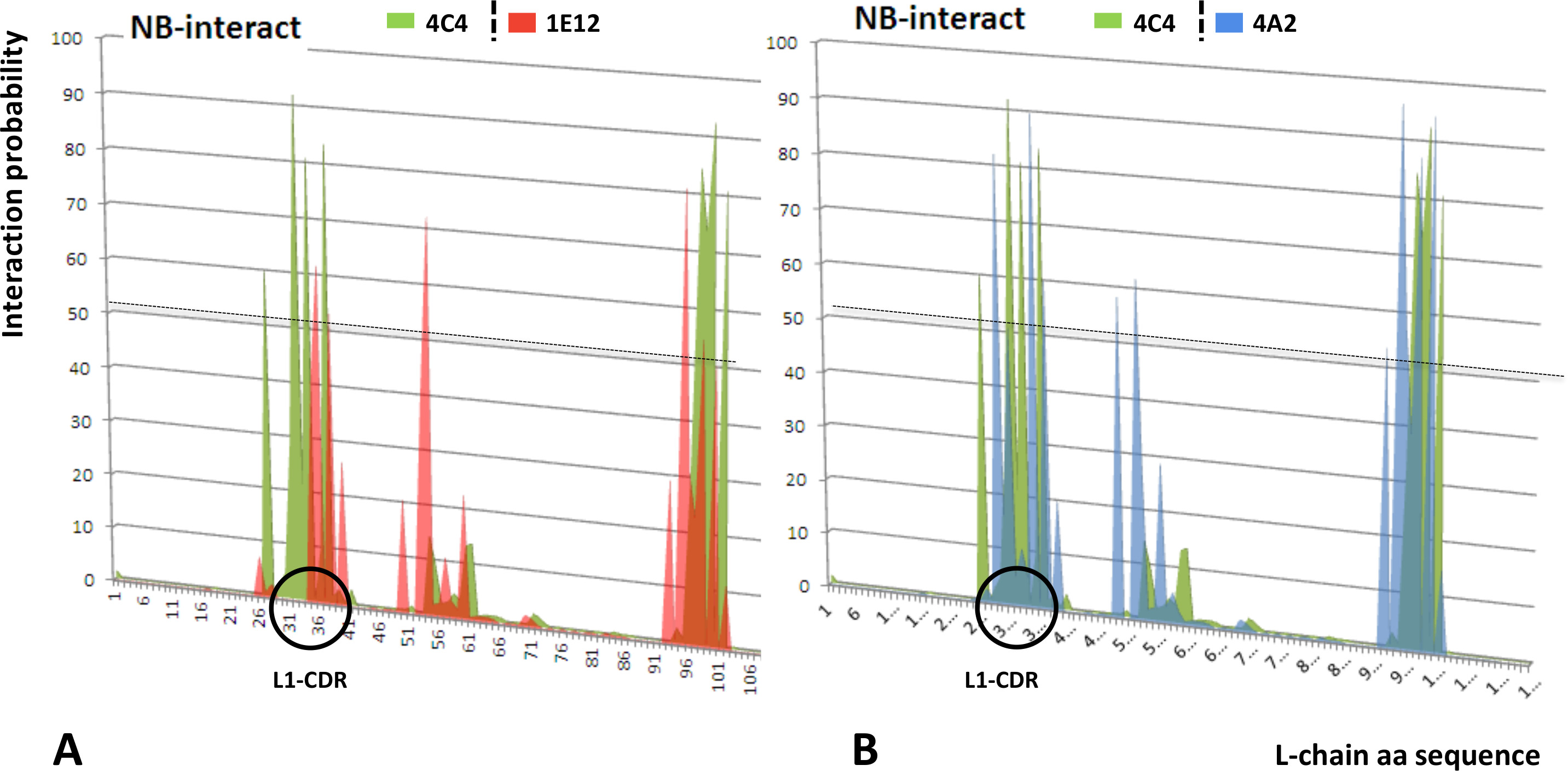

Supplement: Supplementary Figure 3 — L chain-specific mapping of Ag-Ab interaction probability. Non-bonded (NB) interaction probability data per aa residues of the L chains are depicted. The reference 4C4 mAb (green) plot is superposed to 4A2 mAb (blue; A) and 1E12 mAb (red; B) plots. [file Image_3.JPEG]

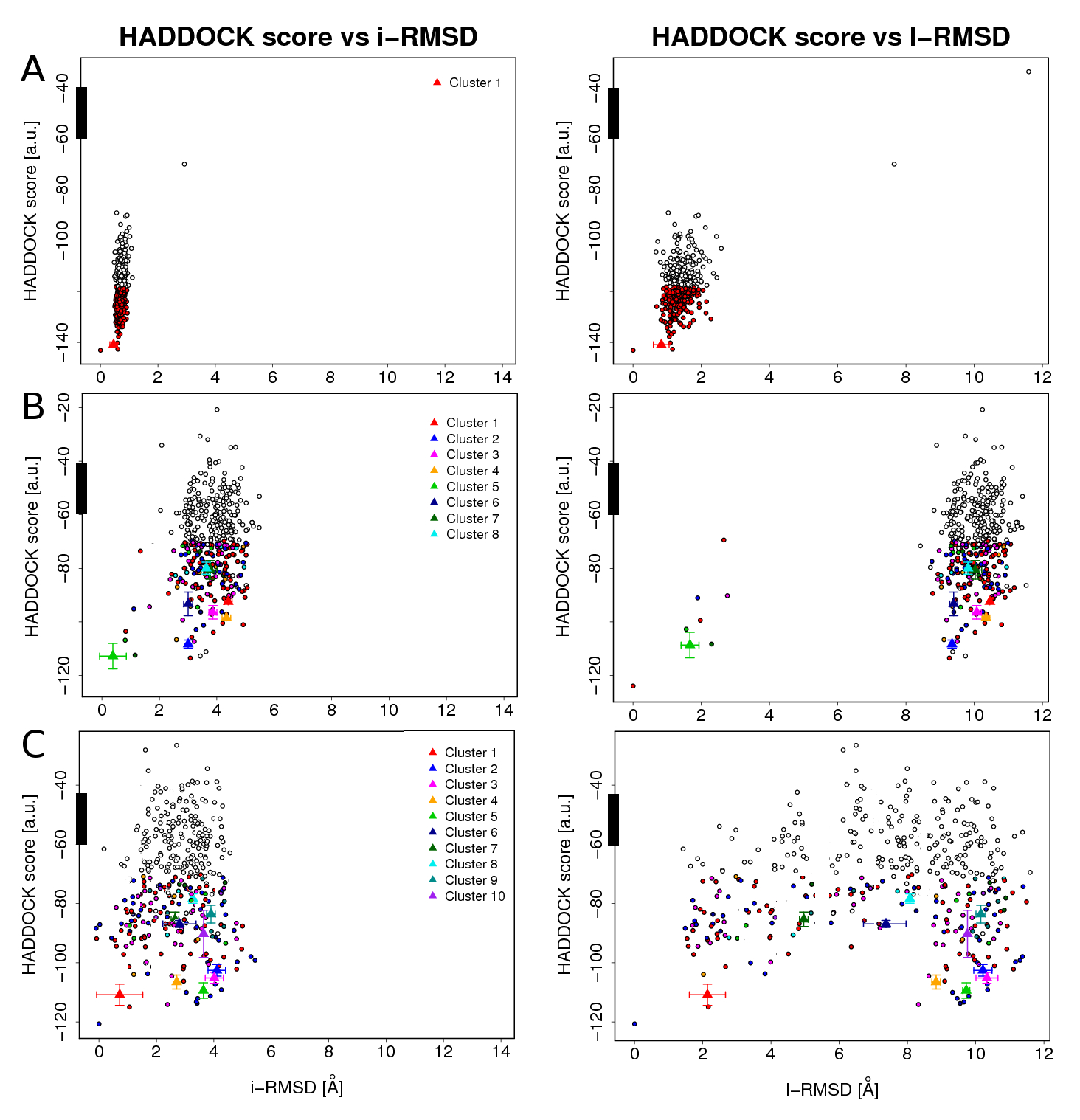

Supplement: Supplementary Figure 4 — Graphic output from the result page of the HADDOCK web server relative to three different molecular docking experiments. The panels display variable docking clusters obtained from 3 different docking inputs of the same FMDV peptide Ag. Single-state Ag input (A) and two different multiple-state Ag input molecular ensembles obtained after 10 (B) or 50 (C) x,y,z coordinates subtle perturbations cycles. The graphics are based on water-refined models generated by HADDOCK. The clusters (indicated in color in the graphs) are calculated based on the interface-ligand RMSDs assessed by HADDOCK, with the interface defined automatically according to all observed contacts. The various FCC, i-RMSD, and l-RMSD structural analyses are made with the best HADDOCK model (the one with the lowest HADDOCK score). Interface-RMSD (i-RMSD) calculated on the backbone (CA, C, N, O, P) atoms of all residues involved in intermolecular contact using a 10Å cutoff. Ligand-RMSD (l-RMSD) calculated on the backbone atoms (CA, C, N, O, P) of all (N > 1) molecules after fitting on the backbone atoms of the first (N = 1) molecule. The average values are calculated on the best 4 structures of each cluster (based on the HADDOCK score). Cluster averages and standard deviations are indicated by colored dots with associated error bars. [file Image_4.JPEG]

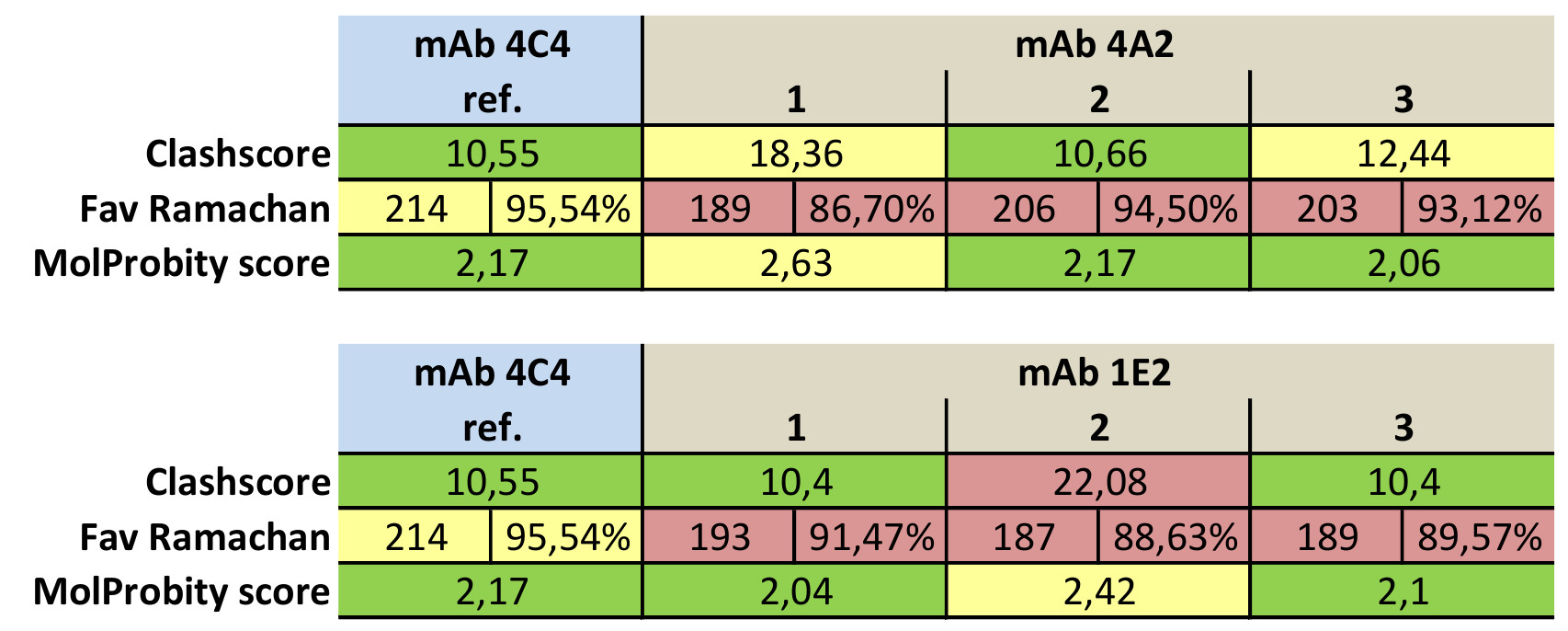

Supplement: Supplementary Figure 5 — Quality scores for Ab molecular models. The scoring, according to MolProbity, includes three metrics (cells in blue) with values that improve according to a scale of colors from red to green. The tables reflect data from 3 models for each of the mAbs 4A2 and 1E12, as well as data for the reference mAb 4C4. [file Image_5.JPEG]

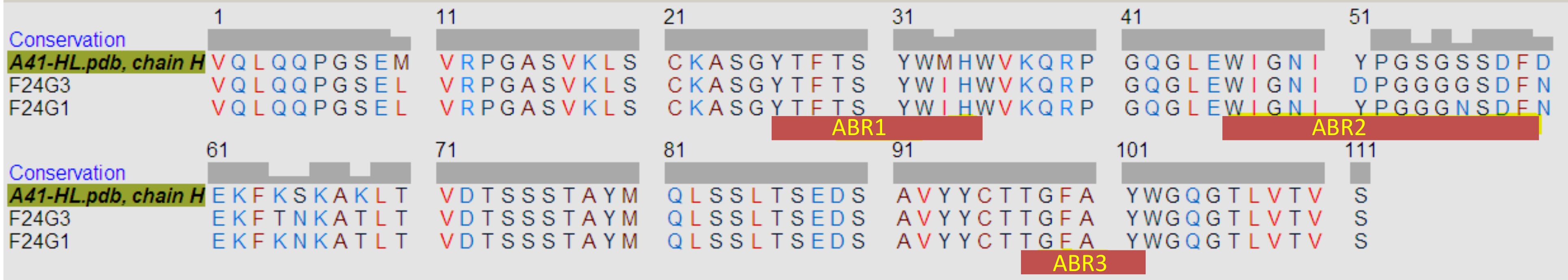

Supplement: Supplementary Figure 6 — Multiple sequence alignment for anti-A24 cruzeiro FMDV mAbs. H-chain aa. sequence alignment between 4A2 mAb (seq. A41-HL) and 2 mAbs developed (F24G3 and G1) against the same FMDV strain. The red boxes indicate CDR1 regions. [file Image_6.JPEG]
