# Supplementary material for: Functional and in silico Characterization of Neutralizing Interactions Between Antibodies and the Foot-and-Mouth Disease Virus Immunodominant Antigenic Site
Source: Front Vet Sci. 2021 May 7;8:554383. doi: 10.3389/fvets.2021.554383 (PMC8137985; doi:10.3389/fvets.2021.554383)
Supplement: Supplementary Table 1 — List of mutant peptides. Mutations and RGD motifs are bold-highlighted. (*)Leu aa usage to contrast the Arg aa prevalence. (**)Pro and Tyr aa usage as chemical structure modifiers for the RGD +1 +4 short helix span. [file Table_1.DOCX]

| **peptide** | **sequence** | **FMDV strain** |
| --- | --- | --- |
| **1** | YAVGGSG**LRGD**MGSLAARVV | * |
| **2** | YA**A**GGSGR**RGD**MGSLAARVV | A/Arg2000/iso104/AY593782 |
| **3** | YAV**N**GSGR**RGD**MGSLAARVV | A/Arg2000/iso104/AY593782 |
| **4** | YAV**S**GSGR**RGD**MGSLAARVV | A/Arg2001/iso93/AY593783 |
| **5** | YAVGG**L**GR**RGD**MGSLAARVV | A/ARG/TLauquen401B/AM180013 |
| **6** | YAVGG**P**GR**RGD**MGSLAARVV | A/ARG/GVillegas00B/AM179992 |
| **7** | YAVGGS**N**R**RGD**MGSLAARVV | A/Arg2000/iso104/AY593782 |
| **8** | YAVGGS**S**R**RGD**MGSLAARVV | A/ARG/JuninI01/AM180017 |
| **9** | YAVGGSGR**RGDL**GSLAARVV | A/ARG/JuninI01/AM180017 |
| **10** | YAVGGSGR**RGDP**GSLAARVV | ** |
| **11** | YAVGGSGR**RGD**M**A**SLAARVV | A/ARG/GVillegas00A/AM179989 |
| **12** | YAVGGSGR**RGD**MG**A**LAARVV | A/Arg2000/iso104/AY593782 |
| **13** | YAVGGSGR**RGD**MGS**Y**AARVV | ** |
| **14 wt** | YAVGGSGR**RGD**MGSLAARVV |  |
| **15 neg** | GFLGAKFR**RGD**PGDMAARVV |  |

**Supplementary Table 1**. List of mutant peptides. Mutations and RGD motifs are bold-highlighted. (*) Leu aa usage to contrast the Arg aa prevalence. (**) Pro and Tyr aa usage as chemical structure modifiers for the RGD +1 +4 short helix span.
